# Stroke and Recurrent Thrombosis of the Coronary Arteries Probably due to Patent Foramen Ovale and Antebrachial Vein Thrombosis in Patient with STEMI – Case Report

**DOI:** 10.15388/Amed.2026.33.1.16

**Published:** 2026-07-22

**Authors:** Anna Konopka, Karolina Marzec, Katarzyna Paschalis-Purtak, Marcin Demkow, Joanna Zalewska, Elżbieta Smaga, Ilona Michałowska

**Affiliations:** 1 Department of Intensive Cardiac Therapy, National Institute of Cardiology, Warsaw, Poland; 2 Department of Hypertension, National Institute of Cardiology, Warsaw, Poland; 3 Department of Coronary and Structural Heart Diseases, National Institute of Cardiology, Warsaw, Poland; 4 Neurology Consultant, National Institute of Cardiology, Warsaw, Poland; 5 Department of Radiology, National Institute of Cardiology, Warsaw, Poland

**Keywords:** stroke, myocardial infarction, insultas, miokardo infarktas

## Abstract

A 74-year-old woman with a history of cryptogenic stroke in 2010 (during hospitalization at the neurological department, possible reasons of stroke were excluded at that time), with less than one year history of paroxysmal atrial fibrillation (AF), which was being treated with apixaban, and also exhibiting hypertension, diabetes type II, hypothyreotocsicosis and obesity, was admitted to our hospital twice. For the first time, the patient was admitted due to ST segment elevation myocardial infarction (STEMI) and an episode of AF. Coronary angiography revealed occlusion of the left circumflex coronary (LCX) artery, and percutaneous coronary intervention (PCI) with stent implantation was performed. Conversions of AF episodes to the sinus rhythm were spontaneous. The patient was discharged home and readmitted once again three days after the discharge. STEMI of the anterior wall and a new episode of AF were then diagnosed. Immediate plain old balloon angioplasty (POBA) with implantation of an additional stent in the left anterior descending coronary artery (LAD) was performed. Sixty minutes after the procedure, the patient developed symptoms of mild right-sided pyramidal syndrome and slight aphasia. The patient’s status was assessed, scoring 3 points according to the *National Institute of Health Stroke Scale* (NIHSS), and the patient was not qualified for thrombolysis and mechanical thrombectomy of the cerebral artery. Four days after manifesting the symptoms of stroke, CT did not reveal any new significant lesions of the brain, and, on the eighth days after the stroke, aphasia almost completely disappeared. A thrombus in the left atrium appendage was excluded, and a patent foramen ovale (PFO) with the left-to-right flow was revealed. Due to the risk of reverse flow as a possible reason of strokes (now and 15 years ago) and recurrent thromboembolization of coronary arteries along with the presence of thromboembolization of the antebrachial vein, successful occlusion of PFO with an Amplatzer Septal Occluder was performed. The patient was discharged home in good condition.

## Abstract (it)

74 metų moteris, kuri 2010 m. patyrė kriptogeninį insultą (tuo metu, kai ji buvo hospitalizuota neurologijos skyriuje, galimos insulto priežastys buvo atmestos), mažiau nei metus serga prieširdžių virpėjimu (PV), jam gydyti skiriama apiksabano, taip pat serga hipertenzija, II tipo cukriniu diabetu, hipotireozine toksikoze ir nutukimu, į mūsų ligoninę buvo paguldyta du kartus. Pirmą kartą moteris paguldyta dėl ST segmento pakilimo miokardo infarkto (STEMI) ir prieširdžių virpėjimo epizodo. Koronarinė angiografija atskleidė, kad yra užsikišusi kairioji apatinė vainikinė arterija (LCX), todėl buvo atlikta perkutaninė koronarinė intervencija (PCI) su stento implantacija. Prieširdžių virpėjimo epizodai savaime perėjo į sinusinį ritmą. Pacientė buvo išrašyta namo, tačiau praėjus trims dienoms po išrašymo ir vėl hospitalizuota. Jai diagnozuotas priekinės sienos STEMI ir naujas prieširdžių virpėjimo epizodas. Nedelsiant atlikta paprastoji balioninė angioplastika (POBA) ir papildomo stento implantacija kairiojoje priekinėje nusileidžiančiojoje vainikinėje arterijoje (LAD). Praėjus 60 minučių po procedūros, pacientei pasireiškė lengvo dešiniojo piramidinio sindromo ir lengvos afazijos simptomai. Pacientės būklė buvo įvertinta 3 balais pagal Nacionalinio sveikatos instituto insulto skalę (NIHSS) ir ji neatitiko reikalavimų trombolizei bei mechaninei smegenų arterijos trombektomijai. Praėjus keturioms dienoms po insulto simptomų, kompiuterinė tomografija neparodė naujų reikšmingų smegenų pažeidimų, o po insulto praėjus 8 dienoms afazija beveik visiškai išnyko. Buvo atmesta trombozė kairiojo prieširdžio priedėlyje ir nustatyta atvira ovalioji anga (PFO) su kraujo srautu iš kairės į dešinę. Dėl atvirkštinio kraujo srauto rizikos kaip galimos insulto priežasties (dabar ir prieš 15 metų) bei pasikartojančios vainikinių arterijų tromboembolijos ir dilbio venos tromboembolijos pacientei buvo sėkmingai atlikta PFO okliuzija naudojant „Amplatzer Septal Occluder“. Būdama geros būklės, pacientė išrašyta namo.

## Introduction

Approximately 25% of ischemic strokes are cryptogenic, and about 16% are further identified as embolic strokes of an undetermined source [1, 2]. In the United States, after a thorough work-up is completed, as many as 30% of ischemic strokes are qualified as cryptogenic, or as an ischemic stroke of an unknown cause [3]. Among patients with embolic strokes of an undetermined source, approximately 25% have a PFO [4]. Other possible mechanisms of cryptogenic stroke include occult paroxysmal atrial fibrillation, substenotic atherosclerosis and hypercoagulability [5]. Transoesophageal echocardiography (TOE) provides the most detailed information about the anatomy and size of a PFO, as well as other high-risk features of stroke.

## Case report

A 74-year-old woman with a history of cryptogenic stroke in 2010, which was confirmed at that time during hospitalization at a neurology department (although the cause of stroke was not detected), with less than one year history of paroxysmal atrial fibrillation (AF), treated with apixaban, and also exhibiting hypertension, diabetes type II, hypothyreotocsicosis and obesity, was admitted to our hospital twice.

**Figure 1:**
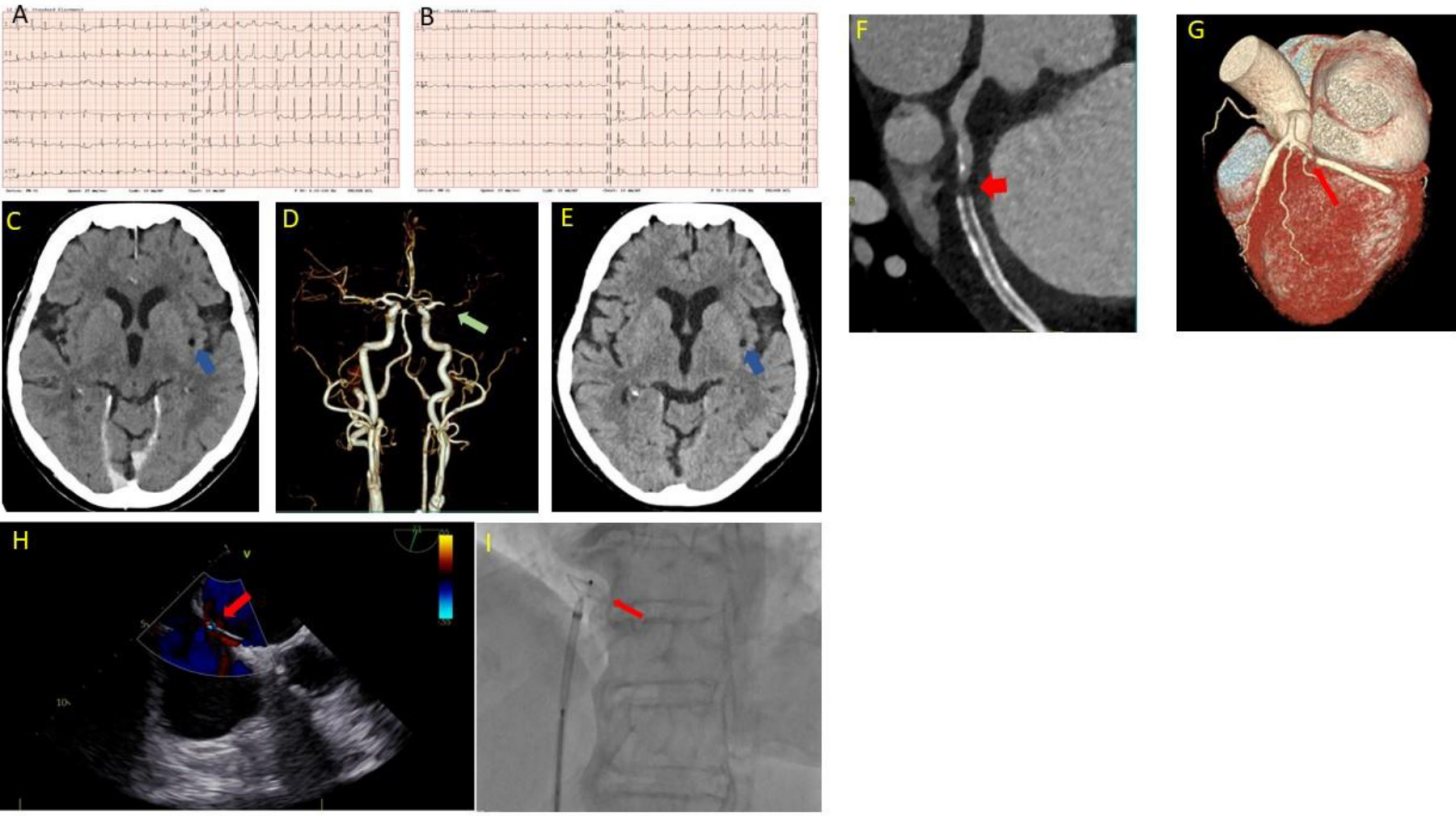
A – ECG at the first admission – inferior MI + AF. B – ECG at the second admission – anterior MI + AF. C – First CNS CT at the beginning of stroke. In left hemisphere focus of old stroke. D – Angio-CT of cerebral arteries – 6 mm defect of contrast of left middle cerebral artery at segments M1, M2 and M3. E – Second CNS CT (4 days after first one) without new lesions. F – Angio-CT of coronary arteries revealed occlusion of LCX in implanted stent. G – Occluded LCX in VRT reconstruction of coronary artery based on angio-CT. H – PFO shown in TEE and Doppler. I – Occluder in PFO. Red arrows – recent lesions and performed procedures, blue arrows – old lesions, green arrow – unknown: recent or old lesion. MI – myocardial infarction, AF – atrial fibrillation, CNS – central nervous system, TEE – transesophageal echocardiography, VRT – volume rendering technique, PFO – patent foramen ovale

First hospitalization: on first admission, ST segment elevation myocardial infarction (STEMI) of the inferior wall accompanied with an episode of atrial fibrillation (AF) was diagnosed (Figure 1A). Coronary angiography revealed an occlusion of the left circumflex coronary (LCX) artery, and *Percutaneous Coronary Intervention* (PCI) with stent implantation was performed, and TIMI 3 was achieved. The procedure was complicated with cardiac arrest (asystole), probably due to the contrast sensitization. After brief resuscitation, ROSC was achieved. Four days after the initial procedure, PCI of 90% narrowed *Left Anterior Descending* (LAD) coronary artery with a stent type DES implantation and paclitaxel eluting balloon angioplasty was performed. In the following days of hospitalization, frequent conversions from AF into the sinus rhythm and from the sinus into AF were observed. After the myocardial infarction, echocardiography revealed left ventricular ejection fraction as 50%. On the 9^th^ day of hospitalization, the patient, in good general condition, while on triple antithrombotic therapy (clopidogrel, aspirin, apixaban), was discharged home.

Second hospitalization: three days after the discharge, STEMI of the anterior wall and a new episode of AF (Figure 1B) were diagnosed. The patient and her family confirmed strict compliance to taking medications. Coronary angiography demonstrated thrombosis within the stent implanted in the LAD. Immediate *Plain Old Balloon Angioplasty* (POBA) with the implantation of an additional stent was performed, and clopidogrel was switched to ticagrelor. At the end of the procedure, TIMI 3 was achieved. Sixty minutes after the procedure, the patient developed symptoms of mild right-sided pyramidal syndrome and slight aphasia. The patient’s neurological status was assessed as 3 points according to the *National Institute of Health Stroke Scale* (NIHSS). The first CT, performed immediately after the symptoms of stroke, revealed small, hypodense foci in deep brain structures, and, at the level of the radiant crown of the brain, vascular lesions incurred at a different time and a 4mm zone of an old stroke in the external capsule of the left hemisphere was detected (Figure 1 C). Angio-CT of cerebral arteries revealed a 6mm defect of contrast of the left middle cerebral artery (LMCA) at segments M1, M2 and M3 (Figure 1D). Except for this pathology, the examination did not reveal atherosclerotic lesions in the brain and carotid arteries.

A neurological consultant did not qualify the patient for thrombolysis and mechanical thrombectomy of the cerebral artery. Intravenous hydration was applied, and antiplatelet therapy was continued. Enoxaparin due to paroxysmal AF was administered. Four days after the symptoms of stroke, a CT was performed, which did not reveal any new significant lesions of the brain, and, on day 8 after the stroke, aphasia almost completely disappeared. Angio-CT of cerebral arteries was not repeated due to the contrast sensitisation. Moreover, during the subsequent course of hospitalization, due to persistent pain and edema, thrombosis of the right forearm artery and vein was detected. Control angio-CT of coronary arteries showed reocclusion of the LCX stent (Figure 1D and 1E). PCI of the LCX was not repeated due to the unknown time of the occlusion and the risk of thrombosis in other coronary arteries during the procedure. The left ventricular ejection fraction was preserved at a level of 50%. In the farther course of the hospitalization, cancer disease and coagulation disorders were excluded. Transoesophageal echocardiography (TOE) excluded thrombus in the left atrium appendage and revealed a *Patent Foramen Ovale* (PFO) with a left-to-right flow (Figure 1H). The patient was consulted by a Heart Team which consisted of a cardiologist, another cardiologist experienced in shunt closure in congenital heart defects, a neurologist, and a third cardiologist denoted by experience in echocardiography. The patient was qualified for PFO occlusion as a preventive measure of further thromboembolic complications. The indications for this procedure were: a risk of reverse flow by PFO as a possible reason of strokes now and 15 years ago, excluded thrombus on the left side of the heart, a short-lasting history of AF with anticoagulation therapy, excluded other risk factors of thrombus formation, i.e., cancer disease and coagulopathy, excluded atherosclerotic lesions in the brain and carotid arteries, and the presence of a potential embolic source from antebrachial vein thrombosis. A doctor denoted by specialized experience performed a successful occlusion of PFO with an Amplatzer Septal Occluder (Figure 1I). The patient was discharged home in good condition.

Three months after the second hospitalization, the patient was admitted to our hospital for control examinations. The clinical status was excellent, and no thromboembolic events were present. A good neurological and cardiological status was also confirmed in CT of the brain and in the angio-CT of coronary arteries.

## Discussion

The patient received 3 points in NIHSS, which did not indicate thrombolysis or mechanical thrombectomy. Fortunately, despite a revealed 6mm defect of contrast in LMCA at segments M1, M2 and M3 suggesting a thrombus, the symptoms of stroke disappeared shortly. There are two explanations of such a clinical course. First, the stroke symptoms may have resulted from hemodynamic instability during acute occlusion of LAD in the patient with a history of stroke and LMCA, probably occluded in the past. Second, the positive effects of intravenous heparin and a loading dose of ticagrelor administered during the preceding PCI may have facilitated rapid dissolution of the clot in the cerebral artery.

Coincidence of an acute stroke with acute coronary syndrome (ACS) is a rare complication, which varies from 1 to 5%, but with high risk of mortality [6, 7]. The pathogenesis of both diseases is different. In cases of acute stroke, the causes of cerebral artery occlusions are more heterogenous than coronary artery occlusions in ACS which are mainly due to atherosclerotic plague with thrombosis [8]. Ischemic stroke can be provoked during revascularization of coronary arteries as a result of AF, or by clot presence in case of ventricular dysfunction. It was shown that every 5% reduction in EF increases the risk of stroke by 18% [7]. In our patient, AF was not present at the time of PCI, and EF of the left ventricle was preserved and calculated as 50%. In our opinion, the risk of stroke due to such causes in the presently described case was very low.

The source of the eventual clot in the cerebral artery could be also the thrombosis of the right forearm vein. Clots can pass to the left side of the heart by PFO under some special circumstances as, for example, a reduction of the arterial blood pressure or a sudden increaseof the central venous pressure.

Although PFOs are mainly present in young patients with cryptogenic stroke (40–50% of cases registered) and despite the fact that not all PFOs confer the same stroke risk, it was shown nevertheless that a PFO closure was effective in preventing a recurrent ischemic stroke as well as some major vascular events, and that the benefit is greater in patients aged ≥60 years than in younger patients [9].

## Conclusion

Overall, the decision to close the PFO in our patient was controversial, but, finally, after strict exclusion of other possible reasons of stroke, the procedure was successfully performed without complications. In a three-month-long follow-up, no thromboembolic complications recurred, and we hope that the PFO closure is bound to prevent thromboembolic events in the future.
